# Germline Profiling and Molecular Characterization of Early Onset Metastatic Colorectal Cancer

**DOI:** 10.3389/fonc.2020.568911

**Published:** 2020-10-19

**Authors:** Ting Xu, Yinjie Zhang, Jing Zhang, Changsong Qi, Dan Liu, Zhenghang Wang, Yanyan Li, Congcong Ji, Jian Li, Xuan Lin, Ting Hou, Hao Liu, Lu Zhang, Han Han-Zhang, Lin Shen, Xicheng Wang

**Affiliations:** ^1^ Department of Gastrointestinal Oncology, Key Laboratory of Carcinogenesis and Translational Research (Ministry of Education), Peking University Cancer Hospital & Institute, Beijing, China; ^2^ Sichuan Cancer Center, School of Medicine, Sichuan Cancer Hospital and Institute, University of Electronic Science and Technology of China, Chengdu, China; ^3^ Department of Radiation Oncology, Beijing Tiantan Hospital, Capital Medical University, Beijing, China; ^4^ Burning Rock Biotech, Guangzhou, China

**Keywords:** early onset colorectal cancer, susceptibility gene, genomic alternation, prognosis, next generation sequencing

## Abstract

**Background:**

Early onset colorectal cancer (EO CRC) is a heterogeneous colorectal cancer subtype with obvious hereditary tendencies and increasing incidence. We sought to determine the susceptibility genes and molecular characteristics of EO CRC.

**Methods:**

330 EO metastatic CRC (mCRC) (≤55 years) and 110 average-onset (AO) mCRC patients (>55 years) were enrolled. Capture-based targeted sequencing was performed on tumor tissue and paired white blood cells using a sequencing panel of 520 genes. The association between molecular alterations and overall survival (OS) was analyzed.

**Results:**

Of the 330 EO mCRC patients, 31 carried pathogenic or likely pathogenic germline mutations, with 16 of them diagnosed with lynch syndrome. Fifteen patients had germline mutations in non-mismatch repair genes, including four in MUTHY, three in RAD50, one in TP53, and eight in other genes. Twenty-nine genes were recurrently mutated in EO mCRC, including TP53, APC, KRAS, SMAD4, and BRCA2. The majority of genomic alterations were comparable between EO and AO mCRC. EO mCRC patients were more likely to have a high tumor mutation burden (p < 0.05). RNF43, RBM10, TSC, and BRAF V600E mutations were more commonly observed in EO mCRC, while APC, ASXL1, DNMT3B, and MET genes were more commonly altered in AO patients. At the pathway level, the WNT pathway was the only differentially mutated pathway between EO and AO mCRC (p < 0.0001). The wild-type WNT pathway (p = 0.0017) and mutated TGF-*β* pathway (p = 0.023) were associated with unfavorable OS in EO mCRC.

**Conclusions:**

Approximately one in 10 EO mCRC was associated with hereditary tumors. The spectrum of somatic alterations was largely comparable between EO and AO mCRC with several notable differences.

## Introduction

Colorectal cancer (CRC) is the fourth most common malignancy and the second leading cause of tumor-related death worldwide (1). CRC is traditionally considered a malignancy that mostly affects individuals over 50 years old, whereas approximately 10% of CRC is diagnosed in individuals before the age of 50 years (2, 3). The incidence and mortality of colorectal cancer have declined globally due to the implementation of routine screening and the advancement of precise treatments (4). However, the age-specific incidence rate among population aged less than 55 years is increasing annually since mid-1990s (5). And mortality rates of colorectal cancer patients aged 20 to 54 years increased by 1.0% annually from 2004 to 2014 (6). Previous studies have shown that early onset colorectal cancer (EO CRC) is often associated with a later stage at presentation, signet ring histology, distal primary tumors, unfavorable prognosis, and strong inherited predisposition compared with average onset colorectal cancer (AO CRC), suggesting that EO CRC might be a distinctive subtype of colorectal cancer (7, 8).

Hereditary cancer syndromes caused by germline mutations in highly penetrant genes account for approximately 2–5% of colorectal cancers (9). With the advent of next generation sequencing (NGS), more tumor susceptibility genes have been identified in CRC. The prevalence of hereditary syndrome in EO CRC ranges from 10 to 35%, which is higher than that in the AO CRC population (9–11). Inherited colorectal tumors, including hereditary non-polyposis colorectal cancer, adenomatosis coli, and suspected HNPCC, accounted for 38.4% of CRC patients under the age 40 years, 17.1% of patients aged 40–50 years, 10.2% of patients aged 50v55, and only 3.5% of individuals older than 55 (12). Furthermore, it should be noted that inherited predisposition alone cannot fully account for the increasing mortality rates in young CRC patients. Comprehensive molecular characterization is required to advance our understanding of the molecular profile of EO CRC, especially metastatic disease which is more challenging in terms of treatment. However, relevant studies are still limited and conflicting results exist in the published literature. In this study, considering the increasing trend of incidence and mortality rates and marked inherited tendency in patients younger than 55 years, we evaluated both the germline and somatic mutation spectrum of 330 early onset metastatic CRC patients using capture-based targeted sequencing to provide clinically actionable therapeutic information and investigate the genomic differences between Chinese EO CRC and Western EO CRC as well as AO CRC patients. We also aimed to explore the prognostic molecular features of EO mCRC.

## Materials and Methods

### Patients and Sample Collection

We conducted a retrospective study on individuals diagnosed with metastatic CRC before or at the age of 55 years who underwent clinical care at the Peking University Cancer Hospital between Jan 2010 and Dec 2018. We enrolled 330 patients who met the following criteria: 1. Diagnosed with metastatic CRC before or at the age of 55 years; 2. Sufficient archived FFPE tumor tissue and paired white blood cell samples for sequencing. In addition, we also enrolled 110 patients with metastatic AO CRC (diagnosed after the age of 55 years) from Burning Rock Dx database for comparative purposes. Tissue samples *via* biopsy or resection and paired white blood cell samples were also obtained from each AO CRC patient. Electronic medical record and telephone interviews were used to obtain information about demographics, family history, tumor location, histology, treatment history, and survival status. This study was approved by the Ethics Committee of the Peking University Cancer Hospital. Informed consent was obtained from each patient.

### Sequencing Panel

The panel used in our study covered 520 cancer-related genes, including all targets of current standard of care targeted therapies, spanning 1.64 mega bases (Mb) of the human genome (OncoScreen Plus, Burning Rock Biotech, Guangzhou, China). Whole exons from 312 genes and critical exons, introns, and promoter regions of the remaining 208 genes as indicated in **Table S1** were included in our panel. The 98 cancer susceptibility genes included in the germline mutation analysis are marked in bold in **Table S1**.

### DNA Extraction

Genomic DNA was extracted from FFPE samples using the QIAamp DNA FFPE tissue kit (Qiagen, Carlsbad, CA, USA) according to the manufacturer’s instructions. DNA concentrations were measured using the Qubit dsDNA assay (Life Technologies, Carlsbad, CA, USA).

### Capture-Based Targeted Sequencing and Analysis

Library preparation was performed in the College of American Pathologists (CAP)-accredited/Clinical Laboratory Improvement Amendments (CLIA)-certified clinical laboratory of Burning Rock Biotech. A minimum of 50 ng of DNA was required for NGS library construction. The genes indicated in **Table S1** were captured and sequenced using a Nextseq500 sequencer (Illumina, Inc., USA) with pair-end reads.

Sequence data were analyzed using proprietary computational algorithms that were optimized for accurate identification of somatic and germline variants, while discriminating sequencing artifacts from true positive mutations. Variants with a population frequency over 0.1% based on the ExAC, 1,000 Genomes, dbSNP, and ESP6500SI-V2 databases were grouped as SNPs and excluded from further analysis.

### Tumor Mutation Burden Estimation

Tumor mutation burden was computed as the ratio between the total numbers of somatic mutations, including synonymous mutations, detected with the total coding region size of the panel used using the formula below. The coding region size of the panel used was 1.26 Mb for the 520-gene OncoScreen Plus panel, which excluded copy number variations, fusions, large genomic rearrangements, and mutations occurring on the kinase domain of *EGFR* and *ALK*.

$$\text{Tumor}\;\text{mutation}\;\text{burden}\;=\;\frac{\text{mutation}\;\text{count}\;(\text{except}\;\text{for}\;\text{copy}\;\text{number}\;\text{variations}\;\text{and}\;\text{fusion})}{\text{total}\;\text{size}\;\text{of}\;\text{coding}\;\text{region}\;\text{of}\;\text{the}\;\text{panel}\;\text{used}}$$

### Microsatellite Instability Determination

All patients were subjected to MSI testing. The MSI (microsatellite instability) phenotype detection method used was a read-count distribution-based approach. It used the coverage ratio of a specific set of repeat lengths as the main characteristic of each microsatellite locus, and categorized a locus as unstable if the coverage ratio was less than a given threshold. The MSI status of a sample was determined by the percentage of unstable loci in a given sample. The MSI status of a tumor sample could then be determined based on the percentage of length-instable loci, without a paired normal control. A total of 63 marker loci were chosen for categorization. For each marker locus, a read-count histogram was constructed, and the coverage ratio of the reference length set was calculated and compared with the reference threshold. A locus with a coverage ratio less than [mean – 3 × SD] of the reference ratio was considered a length-instable locus. A tumor sample was considered MSI-H (microsatellite instability-high) if more than 40% of the marker loci were length instable, MSS (microsatellite stability) if the percentage of length-instable loci were <15%, or MSI-L if the percentage was between 15 and 40%.

### Colorectal Cancer-Related Gene Pathway Analysis

A total of 47 genes involved in six critical pathways in colorectal cancer were selected based on previous studies (13, 14). The 14 genes involved in the WNT pathway were *APC, CTNNB1, DKK1, DKK2, DKK3, DKK4, LRP5, FZD10, FAM123B, AXIN2, TCF7L2, FBXW7, ARID1A*, and *SOX9*. The eight genes selected to represent the MAPK pathway were *KRAS, NRAS, HRAS, NF1, BRAF, ARAF, RAF1*, and *MAP2K1*. The nine genes for the RTK pathway were *ERBB2, ERBB3, EGFR, FGFR1, MET, KIT, NTRK1, NTRK3*, and *RET*. The genes for the PI3K pathway were *PIK3CA, PTEN, PIK3R1, AKT1, TSC1, TSC2*, and *MTOR*. Additionally, seven genes for the TGF-*β* pathway were chosen, namely, *TGFBR1, TGFBR2, ACVR1B, ACVR2A, SMAD2, SMAD3*, and *SMAD4*. Lastly, for the TP53 pathway, *TP53* and *ATM* were selected.

### Statistical Analysis

Descriptive statistics were used to summarize the demographics, clinicopathologic characteristics, and family history. Chi-square or Fisher’s exact tests were performed to compare categorical variables. Survival data were assessed by Kaplan–Meier estimates, and comparisons were made with log-rank tests. A p value < 0.05 was considered statistically significant. All analyses were done using R software, version 3.6.1.

## Results

### Patient Characteristics

From 2010 to 2018, 330 EO mCRC patients diagnosed before or at the age of 55, and 110 patients with AO mCRC (diagnosed after the age of 55) were enrolled in this study. **Table S2** summarizes the demographics and clinicopathological characteristics of early onset patients. The median age for the EO cohort was 45 years (ranging from 13 to 55) (**Figure S1**). The cohort was 54.5% male and 45.5% female. A majority of them (288/330, 87.3%) were diagnosed with adenocarcinoma, while 38 (11.5%) and two (0.6%) were diagnosed with mucinous adenocarcinoma and signet ring cell carcinoma, respectively. One hundred and forty-six (44.3%) patients had left-sided colon cancer, 107 (32.4%) had right-sided colon cancer, and the others (23.3%) had rectal cancer. Ninety-three (28.2%) patients had a family history of any kind of cancer. For the AO CRC cohort, the median age was 64 years (ranging from 56 to 84). Seventy of them (63.6%) were males, and the remaining 40 (36.4%) were females. Of the eighty patients with information in their case history on the primary tumor location, 15 (18.8%) were located in the left side of the colon, 22 (27.5%) had right-sided colon cancer, and 43 (53.7%) had cancer in the rectum.

### Germline Genetic Feature of EO mCRC Patients

Capture-based targeted sequencing was performed by a CLIA-certified lab to interrogate the germline mutation landscape of EO mCRC patients, with 98 cancer susceptibility genes being analyzed. Among the 330 EO mCRC patients, 33 pathogenic or likely pathogenic germline mutations were detected in 31 patients (31/330, 9.4%), and 974 variants of uncertain significance (VUS) in 91 genes were found (**Figure S2**). The germline mutation frequencies of patients younger than 35 years, 35–50 years, and 50–55 years were 22.2%, 7.0%, and 7.9%, respectively. Among the 31 patients, 16 were MSS, and the remaining 15 were MSI-H. Sixteen patients (4.8% of the entire cohort, 52% of patients with pathogenic or likely pathogenic germline mutations) had mutations in one of the DNA mismatch repair (MMR) genes (six in MLH1, three in MSH2, two in PMS2 and five in MSH6). Hence, they had been diagnosed with Lynch Syndrome (LS). One LS patient carrying an MSH6 c.3416del (p. G1139fs) germline mutation showed MSS. Of the 16 patients with LS, six patients had at least one first-degree relative with cancer, and four patients had at least one second-degree relative with cancer. In addition to the MMR genes, other frequently mutated genetic susceptibility genes included but were not limited to, *MUTYH* (*n = 4*, 1.2% of the entire cohort, 12% of patients with germline mutations), *RAD50* (*n = 3*, 0.9% of the entire cohort, 9% of patients with germline mutations), *TP53* (*n = 1*, 0.3% of the entire cohort, 3% of patients with germline mutations) and *FANCL* (*n = 1*, 0.3% of the entire cohort, 3% of patients with germline mutations) (**Table 1**). No individual was found to carry pathogenic or likely pathogenic germline mutations in APC or POLD1/POLE in this cohort.

**Table 1:** Germline Mutations Identified and Associated Syndromes.

Gene	Mutation	Associated hereditary syndrome and cancer type	Patients with Mutation, No. (%)
*MLH1*	p.L296* p.A723fs c.545+1G>A p.L559R p.L525fs p.K618del	Lynch syndrome	16 (4.8%)
*MSH2*	p.R406*, p.S129fs p.G204*		3 (0.9%)
*PMS2*	c.2174+1G>A p.R151fs		2 (0.6%)
*MSH6*	p.S156* p.S156* p.G1139fs p.R1076C p.K1009fs		5 (1.5%)
Monoallelic MUTHY	p.Q414* p.G396D p.Q187* p.Y104*	MUTYH-associated polyposis	4 (1.2%)
*RAD501,2*	p.E723fs p.H1269fs p.E723fs	Nijmegen breakage like syndrome Breast cancer	3 (0.9%)
*NBN*	p.R89*	Nijmegen breakage syndrome, Breast cancer, prostate cancer	1 (0.3%)
*RAD51C*	p.W305*	Breast cancer, gastric cancer, ovarian cancer, prostate cancer	1 (0.3%)
*SDHA*	p.M1?	Gastrointestinal stromal tumors, renal cell carcinoma, paraganglioma, pheochromocytoma	1 (0.3%)
*XRCC2*	p.L14P	Breast cancer	1 (0.3%)
*TP53*	p.M246T	Li-Fraumeni syndrome	1 (0.3%)
*ATR*	p.V316fs	Prostate cancer, cutaneous telangiectasia, and cancer syndrome	1 (0.3%)
*ERCC4*	p.C723*	Breast cancer	1 (0.3%)
*FANCD2*	N1378fs	Breast cancer, head and neck squamous cell carcinoma	1 (0.3%)
*FANCI*	p.Q1277*	Squamous cell carcinomas, Fanconi anemia	1 (0.3%)
*FANCL*	p.F253fs	Squamous cell carcinomas, Fanconi anemia	1 (0.3%)

We next compared the germline mutation spectrum of Chinese EO mCRC patients with a published Western cohort consisting of 430 Caucasian patients diagnosed with CRC before the age of 50 (11). Two hundred and twenty-six patients who were diagnosed before or at the age of 50 from our cohort were included in this comparative analysis. The prevalence of hereditary syndrome 11.1% (n = 25) in our cohort, which was significantly lower than the Western cohort (18%) (p = 0.018). Within hereditary cancer patients, the two cohorts had a comparable percentage of LS patients (64% for our cohort *versus* 71% for the Western cohort, p = 0.61). The distribution of germline mutations in MMR genes was also similar, except for *MSH6* (6% for the Western cohort *versus* 22% for the Chinese cohort, p = 0.043). In addition, germline *APC* mutation, which was not observed in our cohort, accounted for 13% of the Western EO cohort patients. Other high and moderate penetrance genes, including *BRCA1, SMAD4* and *CHEK2*, that were not observed in our cohort, were mutated in 16% of the Western hereditary EO CRC patients.

### Somatic Mutation Spectrum of Early Onset Colorectal Cancer Patients

**Figure 1:**
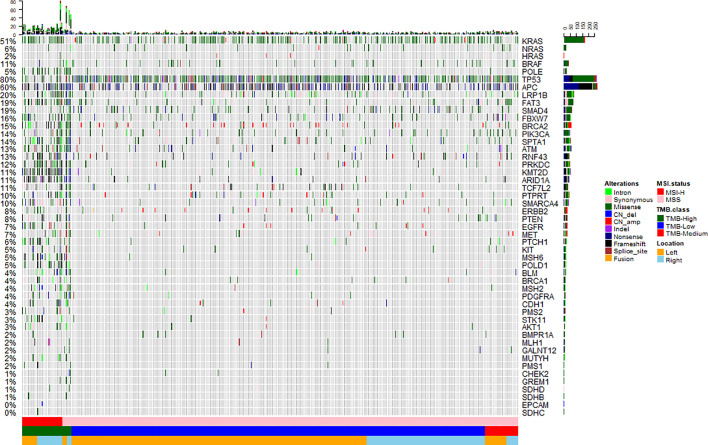
Mutation landscape of early onset metastatic CRC. Heatmap illustrating top 50 genes identified in our study. Each column represents one patient, each row represents an alternation. Upper bars represent the tumor mutation burden. The left bars indicate the frequency of mutated genes. The most frequently mutated were TP53 (80%), APC (60%), KRAS (51%), LRP1B (20%), SMAD4 (19%), and FAT3 (19%). A color key is on the right side.

**Figure 2:**
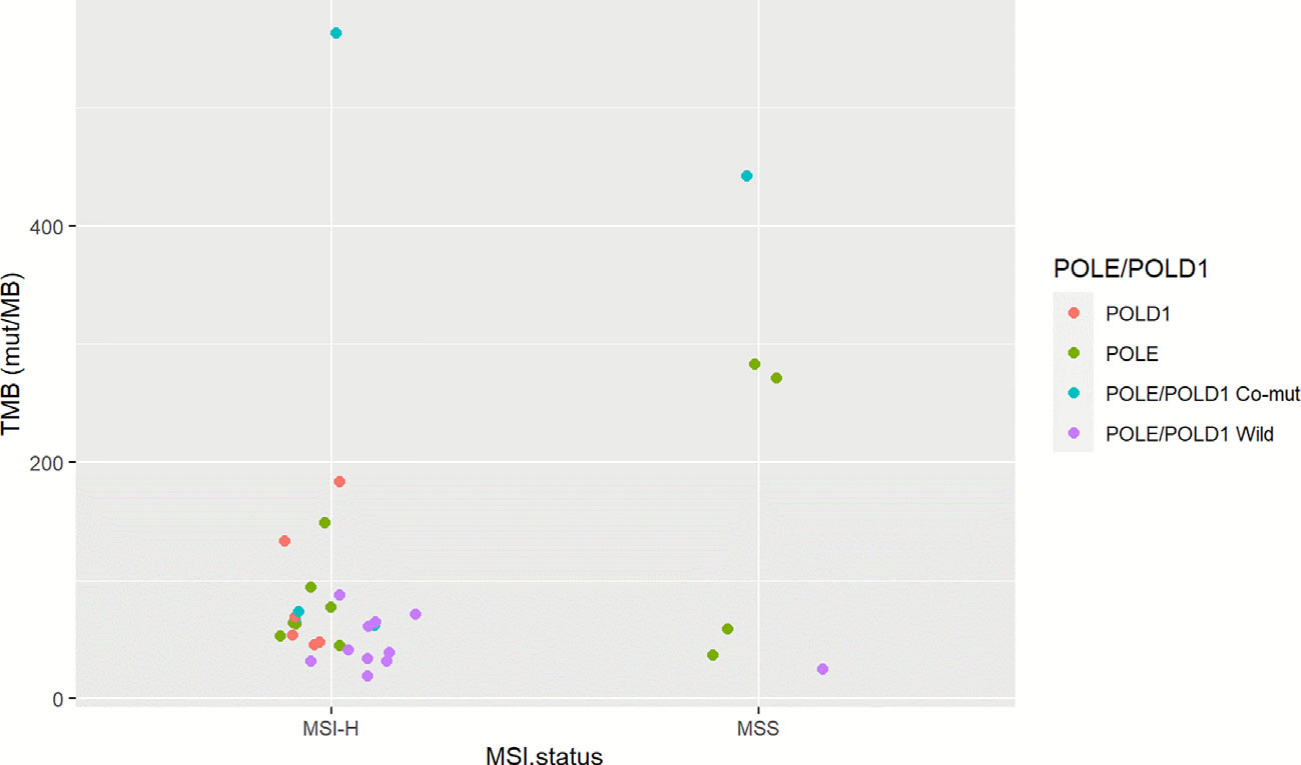
Microsatellite status and POLE/POLD1 mutation of TMB-H EO mCRC cases. The left column illustrates 27 MSI-H cases; the right column indicates six MSS cases. Red represents POLD1 mutation; green indicates patients with POLE mutation; blue indicates patients with POLE-POLD1 co-mutation, and violet indicates patients with non-POLE non-POLD1 mutation.

To investigate the somatic mutation landscape of EO mCRC, DNA extracted from 330 FFPE and paired WBC samples was subjected to next generation sequencing for a panel of 520 cancer related genes and MSI determination. Collectively, we identified 7,096 mutations, spanning 463 genes (21.5 variants per sample on average), including 4,945 single nucleotide variations, 66 insertions or deletions, 453 copy-number amplifications and 10 translocations (**Figure 1**). The median tumor mutational burden (TMB) was 6.7 (range: 0.8–563.5) mutations/Mb across all tumor samples. Thirty-three (10.0%) hypermutated samples (TMB ≥ 20 mutations/Mb) were identified in our cohort, including 27 (8.2%) MSI-H cases and five (1.5%) MSS cases with POLE mutations, (**Figure 2**). Of the five MSS cases with POLE mutations, three were identified with known POLE exonuclease domain mutations (S459F, P286R, and V411L/D275G dual mutation) (**Table S3**). One case with a TMB of 442 per Mb was found to have a POLE S459Y exonuclease domain mutation. This patient also harbored a heterozygous pathogenic MSH6 germline mutation but showed MSS. The remaining one case included a non-hotspot POLE R821C mutation (TMB 59.5 per Mb).

Twenty-nine recurrently mutated genes (mutation frequency ≥ 5%) were identified in the EO mCRC cohort, including *TP53* (80%), *APC* (60%), *KRAS* (51%), *LRP1B* (20%), *SMAD4* (19%) and *FAT3* (19%) (**Figure 1**). Except for TMB-H cases, actionable alterations that may confer sensitivity or resistance to specific targeted therapies were also identified, including 8.8% of tumors with the BRAF V600E mutation, 7.6% with ERBB2 amplification or mutation and 1.5% patients with MET amplification. PTEN and PIK3CA oncogenic mutations were identified in 7.0% and 13.3% of patients, respectively. Targetable fusion mutations were extremely rare and the only two receptor tyrosine kinase fusions were EML4-ALK, GOPC-ROS1. It is worth noting that all BRAF V600E mutant tumors were microsatellite stable in the EO mCRC cohort, in contrast to previous studies. Moreover, seven patients (2.1%) harbored class 3 BRAF mutations with impaired kinase activity.

With the goal of identifying the genomic heterogeneity of EO CRC patients, we selected 47 genes that were related to six critical pathways in CRC and further analyzed the alteration rates of the six driver pathways in EO CRC patients. Mutations were most frequently found in the p53 pathway (85.2%), followed by the MAPK pathway (69.7%), the WNT pathway (69.4%), the TGF*β* pathway (30.0%), the PI3K pathway (27.6%), and RTK genes (27.6%).

### Comparison of the Mutation Landscape of EO and AO mCRC

**Figure 3:**
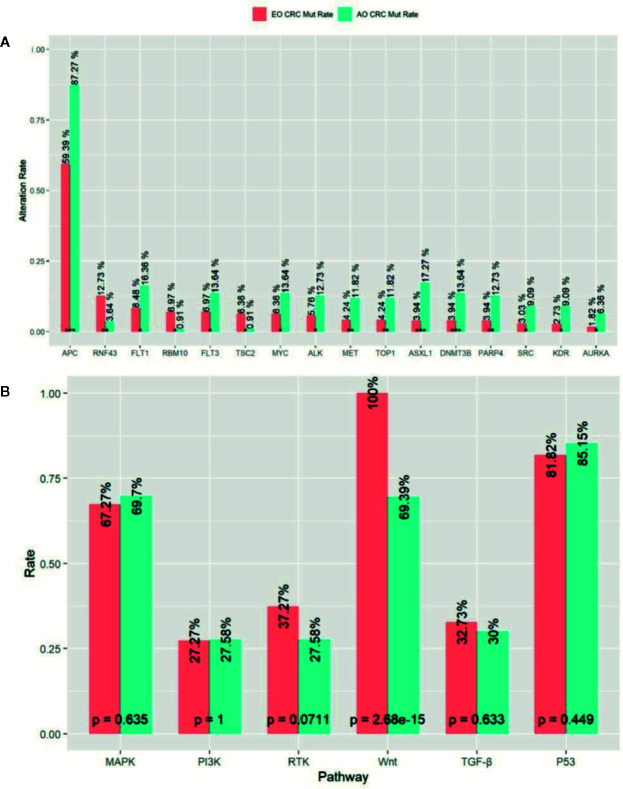
Comparison of Mutation profile between EO mCRC and AO mCRC. **(A)** Alternation rates of differently mutant genes between EO and AO mCRC. **(B)** Pathway mutation rates of EO and AO mCRC. ***p < 0.001; **p < 0.01; *p < 0.05.

In order to investigate whether genomic discrepancies existed between the EO and AO mCRC patients, we also enrolled and analyzed 110 metastatic AO mCRC samples. The median TMB was slightly higher in AO mCRC patients (7.9 mt/Mb). However, the EO mCRC cohort was enriched with TMB-H patients (10.0% *versus* 3.6%, p = 0.046). The mutation rates between the EO mCRC and AO mCRC cohorts were largely similar for most genes of interest. The mutation frequencies of genes which served as resistant biomarkers for anti-EGFR monoclonal antibodies, including *KRAS* (50.3% in EO mCRC *versus* 56.4% in AO mCRC, p = 0.32), *NRAS* (6.1% in EO mCRC *versus* 5.5% in AO mCRC, p = 1), *BRAF* (10.9% in EO CRC *versus* 4.5% in AO CRC, p = 0.056), and *PIK3CA* (13.3% in EO CRC *versus* 12.7% in AO CRC, p = 1) were comparable between the two cohorts. In contrast, the BRAF V600E mutation was more frequently observed in EO mCRC patients (8.8 *vs* 1.8%, p < 0.01). Furthermore, *APC* (p < 0.0001), *ASXL1* (p < 0.0001), *DNMT3B* (p = 0.001), *PARP4* (p = 0.002), *MET* (p = 0.01), *TOP1* (p = 0.01), *KDR* (p = 0.01), *SRC* (p = 0.01), *PAK1* (p = 0.015), *ALK* (p = 0.02), and *MYC* (p = 0.03) alterations were found to be more common in tumor samples from AO mCRC patients. In contrast, there was a significant enrichment of *RNF43* (p = 0.006), *RBM10* (p = 0.01), and *TSC* (p = 0.02) mutations in EO mCRC patients (**Figure 3A**).

We further analyzed the mutation characterizations of the EO and AO mCRC patients at the pathway level. The mutation profile was similar between the EO and AO CRC cohorts except for the WNT pathway (**Figure 3B**). WNT pathway alteration was enriched in AO CRC patients compared with EO CRC patients (100% *versus* 69.4%, p < 0.0001). P53 pathway alteration was found in 85.2% of EO CRC patients and 81.2% of AO CRC patients (p = 0.499), which was more common than the result of previous studies (64–69.0%) (13, 15). There were no significant differences in the mutation frequencies of the MAPK, PI3K, or TGF-*β* pathways between EO and AO CRC patients.

### Molecular Characteristics and Survival

**Figure 4:**
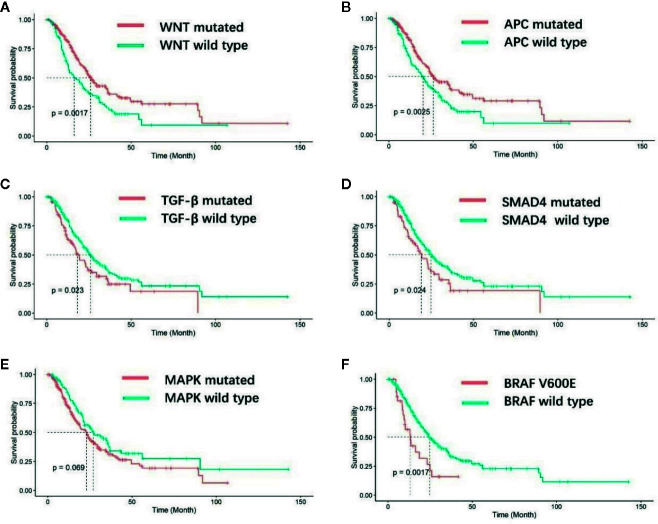
Kaplan–Meier analysis of overall survival for EO mCRC patients with different molecular characteristics. **(A)** Overall survival of WNT pathway wild-type and WNT pathway mutated patients. **(B)** Overall survival of APC gene wild-type and mutated patients. **(C)** Overall survival of TGF-β pathway wild-type and TGF-β pathway mutated patients. **(D)** Overall survival of SMAD4 gene wild-type and SMAD4 mutated patients. **(E)** Overall survival of MAPK pathway wild-type and MAPK pathway mutated patients. **(F)** Overall survival of patients with and without BRAF V600E mutation.

After a median follow-up duration of 16.3 months, 184 deaths occurred at the time of the data cutoff. The median overall survival (OS) was 23.9 months (95% CI 21.0–27.7 m) for the entire EO mCRC cohort. Patients with mutations in the WNT pathway showed a superior prognosis compared with wild type patients (26.0 *versus* 16.4 m p = 0.0017) (**Figure 4A**). Among the fourteen genes in the WNT pathway, APC mutation was found to be associated with better overall survival (25.0 *versus* 19.2 m) (p = 0.0025) (**Figure 4B**). On the contrary, patients with mutations in the TGF-*β* pathway had poorer prognosis in EO mCRC (15.7 *versus* 21.8 m, p = 0.023) (**Figure 4C**), especially SMAD4 gene mutant patients (19.2 *versus* 25.0 m, p = 0.024) (**Figure 4D**). MAPK pathway mutation also indicated a trend of unfavorable overall survival in comparison with MAPK pathway wild type tumors (18.7 *versus* 21.3 m, p = 0.069) (**Figure 4E**). Within MAPK pathway genes, the BRAF V600E mutation was correlated with poorer overall survival compared with BRAF wild type and BRAF non-V600E mutants (13.3 *versus* 24.7 m p = 0.0017) (**Figure 4F**). Neither the p53 pathway nor the PI3K pathway was associated with overall survival in EO mCRC patients.

## Discussion

Herein, we characterized the germline and somatic mutations of early onset metastatic colorectal cancer patients. In this clinic-based cohort of 330 mCRC patients diagnosed at or before the age of 55, 9.4% carried at least one pathogenic or likely-pathogenic germline mutation associated with tumor predisposition as demonstrated using multigene panel sequencing. The germline mutations in cancer susceptibility genes were less commonly seen in our cohort compared with previous studies of EO CRC, which may have partly resulted from racial differences and the late-stage cases chosen for this study. The prevalence of hereditary colorectal cancer elevated markedly in patients diagnosed with mCRC at or before age of 35, which demonstrated that hereditary analyses should be conducted for all patients under 35 without preselection. Notably, of all 31 patients carrying pathogenic or likely-pathogenic germline mutations, only 14 (45.2%) had a family history of malignancy. Moreover, 11 of the 16 LS patients failed to fulfill the Amsterdam II criteria, which strongly suggested that clinical phenotypes and family history were far from sufficient for screening out individuals who need genetic analysis.

The spectrum of tumor susceptibility mutation in our study was different from previous studies. Moderate-penetrance monoallelic MUTYH mutation and APC germline mutations were relatively rare in Chinese EO mCRC. We found 3 patients with scarce compound heterozygotes composed of monoallelic mutations of MUTHY c.53C>T and c.74G>A, which had been previously reported in Japanese polyposis patients and Chinese colorectal cancer patients (16–18). Chen et al. reported that the frequency of this heterozygous haplotype variant allele was statistically higher in CRC patients than in healthy controls (4.35 *versus* 0.87%, p = 0.02). Thus, they concluded that this MUTHY variation is likely to be associated with colorectal cancer susceptibility (17). However, the frequency of monoallelic mutations of MUTHY c.53C>T and c.74G>A in our EO mCRC cohort (0.9%) was very close to that of the healthy controls in Chen’s study (0.87%). Therefore, we classified monoallelic mutations of MUTHY c.53C>T and c.74G>A as variant of uncertain significance in this study. Many of the genes identified in our study have not been demonstrated to be associated with colorectal cancer risk, such as *NBN*, *ATR*, or *RAD50*. Notably, the majority of these patients lacked clinical phenotypes of corresponding hereditary syndromes. Multigene gene panel testing allowed identification of potential susceptibility genes, but additional evidence is needed to establish the relationship between these rare mutations and colorectal cancer.

It has been well-acknowledged that colorectal cancer is a heterogeneous disease with varied molecular mechanisms underlying it (13). Several different classification systems have been established to better understand the biological characteristics of colorectal cancer (19). Another important aspect of our study was that we profiled the unique molecular features of EO mCRC at both the single gene and pathway levels and furthermore evaluated their association with prognosis. The somatic mutation landscape of early onset and average onset mCRC was comparable on the whole. Several critical differences, however, were illustrated in this study. EO mCRC exhibited more TMB-H cases than AO mCRC. The main causes of TMB-H in colorectal cancer include POLE/POLD1 deficiencies and MSI-H resulting from MMR mutation or MLH1 promoter hypermethylation (20). In our EO CRC cohort, 10.0% of patients had a TMB ≥20 mutations/Mb among which 27 exerted MSI-H. The *POLE* gene is mutated in 2.31% of MSS EO mCRC patients, which was close to the mutation frequency in AO mCRC (**Table S3**). A novel exonuclease domain mutation S459Y and a rare non-hotspot mutation R821C were found to be related to hypermutation in our study. There are several previously reported cases reported indicating that POLE R821C is associated with ultra-mutation (21). In previously published studies, the proportion of MSI-H in early onset CRC ranged from 15 to 41.0% (8). Moreover, *POLE/POLD1* mutation was identified in 0.65–12.3% of colorectal cancer patients and was reported to occur more frequently in EO CRC patients (13, 22, 23). The enrichment of *MSI* and *POLE* mutation cases indicated that in EO mCRC, more patients might benefit from immune checkpoint inhibitors, potentially improving the unfavorable prognosis of EO mCRC patients (24, 25).

In our pathway analysis, the WNT pathway was the only differently mutated pathway between EO mCRC and AO mCRC cases. The WNT pathway has long been considered a critical driver pathway for the majority of CRCs. Our study found that EO mCRC patients had a significantly decreased mutation rate in WNT pathway genes, and the presence of mutation in the WNT pathway was associated with better overall survival. APC is one of the key members of the WNT pathway and was previously reported to be mutated in approximately eighty percent of colorectal cancers (13). There was evidence showing that APC mutation was a positive prognostic factor in colorectal cancer, especially in proximal tumors, which was consistent with our results (15, 26, 27). The deregulation of the WNT pathway in EO mCRC patients might account for the unfavorable prognosis to some extent. Thus, more intensive therapies may be applied to EO mCRC patients without WNT pathway mutations to improve their prognosis.

The TGF-*β* pathway was mutant in thirty percent of EO mCRC cases in our study. TGF-*β* signaling plays a key role in tumorigenesis by modulating cell growth, differentiation, and apoptosis, and has an important impact on the tumor microenvironment (28). In the consensus molecular subtype (CMS) classification system of colorectal cancer, TGF-*β* signaling activation was enriched in CMS4 tumors that show upregulation of genes associated with mesenchymal transition or angiogenesis (29). CMS4 tumors also displayed worse overall survival and relapse free survival in comparison with the CMS1-3 subtypes of colorectal cancer (29). TGF-*β* signaling in the tumor microenvironment promotes T-reg cell infiltration and the activation of cancer-associated fibroblasts, which can accelerate tumor progression and impair anti-tumor immunity (30–32). Our study demonstrated that alterations in the TGF-*β* pathway can contribute to aggressive tumor biological characterization and unfavorable outcomes in EO mCRC. Therefore, effective inhibition of the TGF-*β* pathway could be a pivotal strategy in metastatic EO CRC treatment.

MAPK pathway mutation is one of the most vital drivers of colorectal cancer. Although EO mCRC and AO mCRC patients had comparable mutation rates in the MAPK pathway, unexpectedly, the *BRAF* V600E mutation was significantly more common in the EO mCRC cohort. *BRAF* V600E mutation exists in approximately 5–7% of late-stage colorectal cancer as previously reported and was demonstrated to have a significant increase based on patient age in several studies (33–35). In Giulia et al.’s study, no *BRAF* V600E mutations were detected in thirty-three EO CRC patients, which was different from our results (36). Furthermore, *BRAF* V600E mutation is related with *MLH1* promoter methylation, which could lead to microsatellite instability (37). In EO mCRC patients, MSI more frequently resulted from Lynch syndrome, which was usually mutually exclusive from *BRAF* V600E mutation. This could explain the absence of coexistence of MSI-H with *BRAF* V600E mutant tumors in our EO mCRC patients. Our data suggested that *BRAF* V600E could be an important driver mutation in Chinese MSS EO mCRC and could distinguish a particular subtype of EO mCRC with poor prognosis.

There were several limitations in our study. As a single-center and retrospective study, potential regional and selection bias may have existed in the patient population. Considering the low frequency of hereditary cancer syndrome, a larger sample size is required for accurately profiling the germline spectrum of early onset metastatic colorectal cancer. Using multigene panel testing, we found several novel germline mutations that have been rarely reported in hereditary colorectal cancer (*e.g*., *ERCC4*, *SDHA*, and *XRCC2*), but our limited sample size hindered us from determining their penetrance and relationship with colorectal cancer. Although we found various actionable targets in our EO mCRC cohort, the clinical data on corresponding therapies are unavailable to verify the predictive power and clinical significance of these alterations.

In conclusion, 31 of 330 (9.4%) metastatic colorectal cancer patients at or younger than 55 years old carried pathogenic or likely pathogenic cancer susceptibility gene mutations. There were notable differences between the mutation landscape of early onset colorectal cancer and average onset colorectal cancer, which may impact prognosis and response to anti-tumor treatments of early onset colorectal cancer. Identifying hereditary cancer syndrome and therapeutic mutations with next generation sequencing has great practical value for guiding anti-tumor therapies and specialized surveillance of high-risk family members.

## Data Availability Statement

The datasets presented in this study can be found in online repositories. The names of the repository/repositories and accession number(s) can be found below: The National Omics Data Encyclopedia, project ID: OEP001101 (https://www.biosino.org/node/project/detail/OEP001101).

## Ethics Statement

The studies involving human participants were reviewed and approved by Ethics Committee of the Peking University Cancer Hospital. Written informed consent to participate in this study was provided by the participants’ legal guardian/next of kin.

## Author Contributions

XW, TX, and YZ designed the project. JZ, CQ, DL, ZW, YL, CJ, and JL participated in patient selection and data collection. TH, LZ and HH-Z carried out next generation sequencing, analyzed and interpreted the data. XL preformed statistical analysis. TX, XW, and LS wrote the manuscript. All authors contributed to the article and approved the submitted version.

## Funding

This project is supported by National Key R&D Program of China (Grant No. 2017YFC0908200).

## Conflict of Interest

Authors XL, TH, HL, LZ, and HH-Z are employed by the company Burning Rock Biotech.

The remaining authors declare that the research was conducted in the absence of any commercial or financial relationships that could be construed as a potential conflict of interest.

## Abbreviations

AO CRC, average onset colorectal cancer; APC, adenomatous polyposis coli; EO CRC, early onset colorectal cancer; FFPE, formalin fixed paraffin-embedded; LS, Lynch Syndrome; MAPK, mitogen-activated protein kinase; MMR, mismatch repair; MSI-H, microsatellite instability high; MSS, microsatellite stability; NGS, next generation sequencing; OS, overall survival; PI3K, phosphatidylinositol 3-hydroxy kinase; RTK, receptor tyrosine kinase TMB, tumor mutation burden; TGF-*β*, transforming growth factor *β*.

## References

[1] Bray F Ferlay J Soerjomataram I Siegel RL Torre LA Jemal A Global cancer statistics 2018: GLOBOCAN estimates of incidence and mortality worldwide for 36 cancers in 185 countries. CA Cancer J Clin (2018) 68(6):394–424. 30207593 10.3322/caac.21492

[2] Chang DT Pai RK Rybicki LA Dimaio MA Limaye M Jayachandran P Clinicopathologic and molecular features of sporadic early-onset colorectal adenocarcinoma: an adenocarcinoma with frequent signet ring cell differentiation, rectal and sigmoid involvement, and adverse morphologic features. Mod Pathol (2012) 25(8):1128–39. 10.1038/modpathol.2012.61 22481281

[3] Siegel RL Miller KD Fedewa SA Ahnen DJ Meester RGS Barzi A Colorectal cancer statistics, 2017. CA Cancer J Clin (2017) 67(3):177–93. 10.3322/caac.21395 28248415

[4] Arnold M Sierra MS Laversanne M Soerjomataram I Jemal A Bray F Global patterns and trends in colorectal cancer incidence and mortality. Gut. (2017) 66(4):683–91. 10.1136/gutjnl-2015-310912 26818619

[5] Wolf AMD Fontham ETH Church TR Flowers CR Guerra CE LaMonte SJ Colorectal cancer screening for average-risk adults: 2018 guideline update from the American Cancer Society. CA: Cancer J Clin (2018) 68(4):250–81. 10.3322/caac.21457 29846947

[6] Siegel RL Miller KD Jemal A Colorectal Cancer Mortality Rates in Adults Aged 20 to 54 Years in the United States, 1970-2014. Jama. (2017) 318(6):572–4. 10.1001/jama.2017.7630 PMC5817468 28787497

[7] Yantiss RK Goodarzi M Zhou XK Rennert H Pirog EC Banner BF Clinical, pathologic, and molecular features of early-onset colorectal carcinoma. Am J Surg Pathol (2009) 33(4):572–82. 10.1097/PAS.0b013e31818afd6b 19047896

[8] Silla IO Rueda D Rodríguez Y García JL de la Cruz Vigo F Perea J Early-onset colorectal cancer: a separate subset of colorectal cancer. World J Gastroenterol (2014) 20(46):17288–96. 10.3748/wjg.v20.i46.17288 PMC4265586 25516639

[9] Mork ME You YN Ying J Bannon SA Lynch PM Rodriguez-Bigas MA High Prevalence of Hereditary Cancer Syndromes in Adolescents and Young Adults With Colorectal Cancer. J Clin Oncol (2015) 33(31):3544–9. 10.1200/JCO.2015.61.4503 PMC4979241 26195711

[10] Pearlman R Frankel WL Swanson B Zhao W Yilmaz A Miller K Prevalence and Spectrum of Germline Cancer Susceptibility Gene Mutations Among Patients With Early-Onset Colorectal Cancer. JAMA Oncol (2017) 3(4):464–71. 10.1001/jamaoncol.2016.5194 PMC5564179 27978560

[11] Stoffel EM Koeppe E Everett J Ulintz P Kiel M Osborne J Germline Genetic Features of Young Individuals With Colorectal Cancer. Gastroenterology (2018) 154(4):897–905.e1. 29146522 10.1053/j.gastro.2017.11.004 PMC5847426

[12] Fante R Benatti P di Gregorio C De Pietri S Pedroni M Tamassia MG Colorectal carcinoma in different age groups: a population-based investigation. Am J Gastroenterol (1997) 92(9):1505–9. 9317073

[13] Cancer Genome Atlas N Comprehensive molecular characterization of human colon and rectal cancer. Nature (2012) 487(7407):330–7. 10.1038/nature11252 PMC3401966 22810696

[14] Yaeger R Chatila WK Lipsyc MD Hechtman JF Cercek A Sanchez-Vega F Clinical Sequencing Defines the Genomic Landscape of Metastatic Colorectal Cancer. Cancer Cell (2018) 33(1):125–36.e3. 29316426 10.1016/j.ccell.2017.12.004 PMC5765991

[15] Lee DW Han SW Cha Y Bae JM Kim HP Lyu J Association between mutations of critical pathway genes and survival outcomes according to the tumor location in colorectal cancer. Cancer (2017) 123(18):3513–23. 10.1002/cncr.30760 28513830

[16] Yanaru-Fujisawa R Matsumoto T Ushijima Y Esaki M Hirahashi M Gushima M Genomic and functional analyses of MUTYH in Japanese patients with adenomatous polyposis. Clin Genet (2008) 73(6):545–53. 10.1111/j.1399-0004.2008.00998.x 18422726

[17] Chen H Xu L Qi Q Yao Y Zhu M Wang Y A haplotype variation affecting the mitochondrial transportation of hMYH protein could be a risk factor for colorectal cancer in Chinese. BMC Cancer (2008) 8:269. 18811933 10.1186/1471-2407-8-269 PMC2565682

[18] Takao M Yamaguchi T Eguchi H Tada Y Kohda M Koizumi K Characteristics of MUTYH variants in Japanese colorectal polyposis patients. Int J Clin Oncol (2018) 23(3):497–503. 29330641 10.1007/s10147-017-1234-7

[19] Dienstmann R Vermeulen L Guinney J Kopetz S Tejpar S Tabernero J Consensus molecular subtypes and the evolution of precision medicine in colorectal cancer. Nat Rev Cancer (2017) 17(2):79–92. 28050011 10.1038/nrc.2016.126

[20] Sclafani F PD-1 inhibition in metastatic dMMR/MSI-H colorectal cancer. Lancet Oncol (2017) 18(9):1141–2. 10.1016/S1470-2045(17)30512-0 28734760

[21] Campbell BB Light N Fabrizio D Zatzman M Fuligni F de Borja R Comprehensive Analysis of Hypermutation in Human Cancer. Cell. (2017) 171(5):1042–56.e10. 29056344 10.1016/j.cell.2017.09.048 PMC5849393

[22] Guerra J Pinto C Pinto D Pinheiro M Silva R Peixoto A POLE somatic mutations in advanced colorectal cancer. Cancer Med (2017) 6(12):2966–71. 10.1002/cam4.1245 PMC5727326 29072370

[23] Domingo E Freeman-Mills L Rayner E Glaire M Briggs S Vermeulen L Somatic POLE proofreading domain mutation, immune response, and prognosis in colorectal cancer: a retrospective, pooled biomarker study. Lancet Gastroenterol Hepatol (2016) 1(3):207–16. 10.1016/S2468-1253(16)30014-0 28404093

[24] Ganesh K Stadler ZK Cercek A Mendelsohn RB Shia J Segal NH Immunotherapy in colorectal cancer: rationale, challenges and potential. Nat Rev Gastroenterol Hepatol (2019) 16(6):361–75. 10.1038/s41575-019-0126-x PMC7295073 30886395

[25] Overman MJ McDermott R Leach JL Lonardi S Lenz H-J Morse MA Nivolumab in patients with metastatic DNA mismatch repair-deficient or microsatellite instability-high colorectal cancer (CheckMate 142): an open-label, multicentre, phase 2 study. Lancet Oncol (2017) 18(9):1182–91. 10.1016/S1470-2045(17)30422-9 PMC6207072 28734759

[26] Schell MJ Yang M Teer JK Lo FY Madan A Coppola D A multigene mutation classification of 468 colorectal cancers reveals a prognostic role for APC. Nat Commun (2016) 7:11743–. 10.1038/ncomms11743 PMC4912618 27302369

[27] Jorissen RN Christie M Mouradov D Sakthianandeswaren A Li S Love C Wild-type APC predicts poor prognosis in microsatellite-stable proximal colon cancer. Br J Cancer (2015) 113(6):979–88. 10.1038/bjc.2015.296 PMC4578087 26305864

[28] Itatani Y Kawada K Sakai Y Transforming Growth Factor-beta Signaling Pathway in Colorectal Cancer and Its Tumor Microenvironment. Int J Mol Sci (2019) 20(23):5822. 10.3390/ijms20235822 PMC6929101 31756952

[29] Guinney J Dienstmann R Wang X de Reynies A Schlicker A Soneson C The consensus molecular subtypes of colorectal cancer. Nat Med (2015) 21(11):1350–6. 10.1038/nm.3967 PMC4636487 26457759

[30] Shin N Son GM Shin D-H Kwon M-S Park B-S Kim H-S Cancer-Associated Fibroblasts and Desmoplastic Reactions Related to Cancer Invasiveness in Patients With Colorectal Cancer. Ann Coloproctol (2019) 35(1):36–46. 30879282 10.3393/ac.2018.09.10 PMC6425246

[31] Yamada N Kuranaga Y Kumazaki M Shinohara H Taniguchi K Akao Y Colorectal cancer cell-derived extracellular vesicles induce phenotypic alteration of T cells into tumor-growth supporting cells with transforming growth factor-β1-mediated suppression. Oncotarget. (2016) 7(19):27033–43. 10.18632/oncotarget.7041 PMC5053630 27081032

[32] Hawinkels LJ Paauwe M Verspaget HW Wiercinska E van der Zon JM van der Ploeg K Interaction with colon cancer cells hyperactivates TGF-beta signaling in cancer-associated fibroblasts. Oncogene (2014) 33(1):97–107. 23208491 10.1038/onc.2012.536

[33] Berg M Danielsen SA Ahlquist T Merok MA Ågesen TH Vatn MH DNA sequence profiles of the colorectal cancer critical gene set KRAS-BRAF-PIK3CA-PTEN-TP53 related to age at disease onset. PloS One (2010) 5(11):e13978–e. 10.1371/journal.pone.0013978 PMC2980471 21103049

[34] Willauer AN Liu Y Pereira AAL Lam M Morris JS Raghav KPS Clinical and molecular characterization of early-onset colorectal cancer. Cancer (2019) 125(12):2002–10. 10.1002/cncr.31994 PMC6583775 30854646

[35] Kawazoe A Shitara K Fukuoka S Kuboki Y Bando H Okamoto W A retrospective observational study of clinicopathological features of KRAS, NRAS, BRAF and PIK3CA mutations in Japanese patients with metastatic colorectal cancer. BMC Cancer (2015) 15:258–. 10.1186/s12885-015-1276-z PMC4393591 25886136

[36] Magnani G Furlan D Sahnane N Reggiani Bonetti L Domati F Pedroni M Molecular Features and Methylation Status in Early Onset (</=40 Years) Colorectal Cancer: A Population Based, Case-Control Study. Gastroenterol Res Pract (2015) 2015:132190. 26557847 10.1155/2015/132190 PMC4629034

[37] Kane MF Loda M Gaida GM Lipman J Mishra R Goldman H Methylation of the hMLH1 promoter correlates with lack of expression of hMLH1 in sporadic colon tumors and mismatch repair-defective human tumor cell lines. Cancer Res (1997) 57(5):808–11. 9041175

