# Supplementary figures and images for: Germline Profiling and Molecular Characterization of Early Onset Metastatic Colorectal Cancer
Source: Front Oncol. 2020 Oct 19;10:568911. doi: 10.3389/fonc.2020.568911 (PMC7604404; doi:10.3389/fonc.2020.568911)

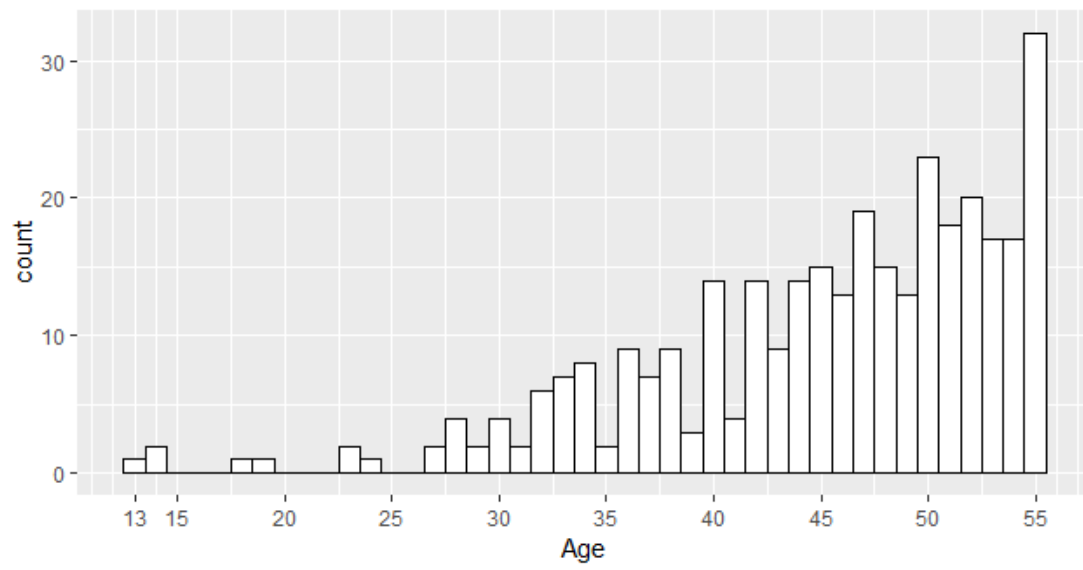

Figure S1 Age distribution of 330 EO mCRC

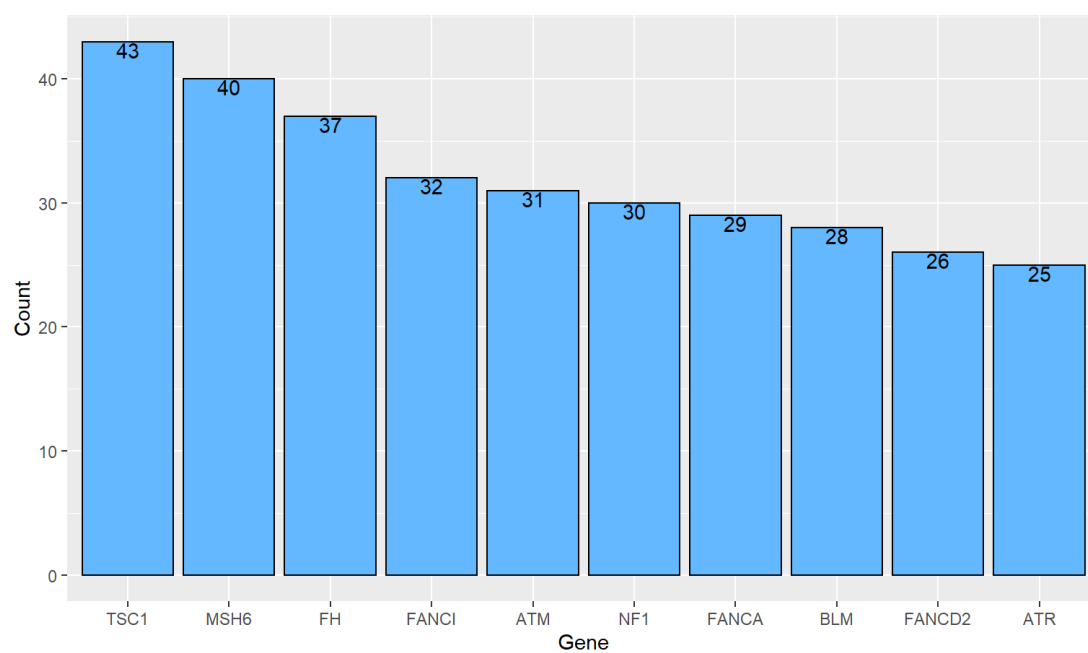

Figure S2 Top 10 VUS genes identified in 330 EO CRC patients

Supplement: Supplementary file 1 [file Presentation_1.pdf]
